# Treatment Outcomes and Cost-Effectiveness of Shifting Management of Stable ART Patients to Nurses in South Africa: An Observational Cohort

**DOI:** 10.1371/journal.pmed.1001055

**Published:** 2011-07-19

**Authors:** Lawrence Long, Alana Brennan, Matthew P. Fox, Buyiswa Ndibongo, Imogen Jaffray, Ian Sanne, Sydney Rosen

**Affiliations:** 1 Health Economics and Epidemiology Research Office, Wits Health Consortium, Johannesburg, South Africa; 2 Faculty of Health Sciences, University of the Witwatersrand, Johannesburg, South Africa; 3 Center for Global Health and Development, Boston University, Boston, Massachusetts, United States of America; 4 Department of Epidemiology, Boston University School of Public Health, Boston University, Boston, Massachusetts, United States of America; 5 Right to Care, Johannesburg, South Africa; Médecins Sans Frontières, South Africa

## Abstract

**Background:**

To address human resource and infrastructure shortages, resource-constrained countries are being encouraged to shift HIV care to lesser trained care providers and lower level health care facilities. This study evaluated the cost-effectiveness of down-referring stable antiretroviral therapy (ART) patients from a doctor-managed, hospital-based ART clinic to a nurse-managed primary health care facility in Johannesburg, South Africa.

**Methods and Findings:**

Criteria for down-referral were stable ART (≥11 mo), undetectable viral load within the previous 10 mo, CD4>200 cells/mm^3^, <5% weight loss over the last three visits, and no opportunistic infections. All patients down-referred from the treatment-initiation site to the down-referral site between 1 February 2008 and 1 January 2009 were compared to a matched sample of patients eligible for down-referral but not down-referred. Outcomes were assigned based on vital and health status 12 mo after down-referral eligibility and the average cost per outcome estimated from patient medical record data.

The down-referral site (*n* = 712) experienced less death and loss to follow up than the treatment-initiation site (*n* = 2,136) (1.7% versus 6.2%, relative risk = 0.27, 95% CI 0.15–0.49). The average cost per patient-year for those in care and responding at 12 mo was US$492 for down-referred patients and US$551 for patients remaining at the treatment-initiation site (*p*<0.0001), a savings of 11%. Down-referral was the cost-effective strategy for eligible patients.

**Conclusions:**

Twelve-month outcomes of stable ART patients who are down-referred to a primary health clinic are as good as, or better than, the outcomes of similar patients who are maintained at a hospital-based ART clinic. The cost of treatment with down-referral is lower across all outcomes and would save 11% for patients who remain in care and respond to treatment. These results suggest that this strategy would increase treatment capacity and conserve resources without compromising patient outcomes.

*Please see later in the article for the Editors' Summary*

## Abstract 2

Lawrence Long and colleagues report that “down-referring” stable HIV patients from a doctor-managed, hospital-based ART clinic to a nurse-managed primary health facility provides good health outcomes and cost-effective treatment for patients.

## Editors' Summary

**Background:**

AIDS has killed more than 25 million people since 1981, and about 33 million people are now infected with HIV, the virus that causes AIDS. Because HIV destroys immune system cells, which leaves infected individuals susceptible to other infections, early in the AIDS epidemic, most HIV-infected people died within ten years of infection. Then, in 1996, antiretroviral therapy (ART), which can keep HIV in check for many years, became available. For people living in developed countries, HIV infection became a chronic condition, but people in developing countries were not so lucky—ART was prohibitively expensive and so a diagnosis of HIV infection remained a death sentence in many regions of the world. In 2003, this situation was declared a global health emergency, and governments, international agencies, and funding bodies began to implement plans to increase ART coverage in developing countries. As a result, nowadays, more than a third of people in low- and middle-income countries who need ART are receiving it.

**Why Was This Study Done?:**

Unfortunately, shortages of human resources in developing countries are impeding progress toward universal ART coverage. In sub-Saharan Africa, for example, where two-thirds of all HIV-positive people live, there are too few doctors to supervise all the ART that is required. Various organizations are therefore encouraging a shift of clinical care responsibilities for people receiving ART from doctors to less highly trained, less expensive, and more numerous members of the clinical workforce. Thus, in South Africa, plans are underway to reduce the role of hospital doctors in ART and to increase the role of primary health clinic nurses. One specific strategy involves “down-referring” patients whose HIV infection is under control (“stable ART patients”) from a doctor-managed, hospital-based ART clinic to a nurse-managed primary health care facility. In this observational study, the researchers investigate the effect of this strategy on treatment outcomes and costs by retrospectively analyzing data collected from a cohort (group) of adult patients initially treated by doctors at the Themba Lethu Clinic in Johannesburg and then down-referred to a nearby primary health clinic where nurses supervised their treatment.

**What Did the Researchers Do and Find?:**

Patients attending the hospital-based ART clinic were invited to transfer to the down-referral site if they had been on ART for at least 11 months and met criteria that indicated that ART was controlling their HIV infection. Each of the 712 stable ART patients who agreed to be down-referred to the primary health clinic was matched to three patients eligible for down-referral but not down-referred (2,136 patients), and clinical outcomes and costs in the patient groups were compared 12 months after down-referral eligibility. At this time point, 1.7% of the down-referred patients had died or had been lost to follow up compared to 6.2% of the patients who continued to receive hospital-based ART. The average cost per patient-year for those in care and responding at 12 months was US$492 for down-referred patients but US$551 for patients remaining at the hospital. Finally, the down-referral site spent US$509 to produce a patient who was in care and responding one year after down-referral on average, whereas the hospital spent US$602 for each responding patient. Thus, the down-referral strategy (nurse-managed care) was more cost-effective than continued hospital treatment (doctor-managed care).

**What Do These Findings Mean?:**

These findings indicate that, at least for this pair of study sites, the 12-month outcomes of stable ART patients who were down-referred to a primary health clinic were as good as or better than the outcomes of similar patients who remained at a hospital-based ART clinic. Moreover, the down-referral strategy saved 11% of costs for patients who remained in care and responded to treatment and appeared to be cost-effective, although additional studies are needed to confirm this last finding. Because this is an observational study (that is, patients eligible for down-referral were not randomly assigned to hospital or primary care facility treatment), it is possible that some unknown factor was responsible for the difference in outcomes between the two patient groups. Nevertheless, these results suggest that the down-referral strategy tested in this study could increase ART capacity and conserve resources without compromising patient outcomes in South Africa and possibly in other resource-limited settings.

**Additional Information:**

Please access these Web sites via the online version of this summary at http://dx.doi.org/journal.pmed.pmed.1001055.

- This study is further discussed in a *PLoS Medicine* Perspective by Ford and Mills
- The US National Institute of Allergy and Infectious Diseases provides information on HIV infection and AIDS
- HIV InSite has comprehensive information on HIV/AIDS
- Information is available from Avert, an international AIDS charity, on many aspects of HIV/AIDS, including information on HIV and AIDS in Africa and on universal access to AIDS treatment (in English and Spanish)
- The World Health Organization provides information about universal access to AIDS treatment, including its 2010 progress report (in English, French and Spanish)
- Right to Care, a non-profit organization that aims to deliver and support quality clinical services in Southern Africa for the prevention, treatment, and management of HIV, provides information on down-referral

## Introduction

The rapid expansion of large-scale provision of care and treatment for HIV/AIDS in sub-Saharan Africa has placed tremendous pressure on the region's human resources. As a result, the World Health Organization, other international agencies, and national governments are actively encouraging the shifting of clinical care responsibilities to lesser trained, less expensive, and generally less scarce cadres of the clinical workforce [1].

In South Africa, a major component of this “task shifting” strategy is to reduce the role of doctors in managing patients on antiretroviral therapy (ART), in favor of primary health care nurses (PHCNs). Unlike in many countries in the region, South Africa's national treatment program has been largely managed by doctors. Nurses have not been authorized to dispense antiretroviral drugs (ARVs), initiate patients on treatment, or conduct medical examinations in lieu of doctors. The number of accredited sites authorized to provide ART has thus been limited by the availability of doctors. Since most primary health care facilities are staffed entirely by nurses, most accredited ART sites have been located at hospitals rather than primary care clinics.

There is a small but expanding body of evidence from sub-Saharan Africa demonstrating that nurses managing ART patients at primary health care facilities can achieve very good outcomes. A recent systematic review of task-shifting in Africa identified seven cohort studies of nurse-initiated and/or nurse-managed adult treatment [2]. Those that reported outcomes generally showed nurses achieved comparable or superior results to doctor-led treatment in the same general setting, but only one undertook a direct comparison of doctor-managed versus nurse-managed treatment of similar patient populations [3]. Primary health care facilities achieved better outcomes than hospitals in a recent study from South Africa, but patients were treated by doctors at both levels [4]. A prospective controlled evaluation in Swaziland found equally good outcomes amongst stable HIV patients managed by nurses in a primary care facility compared to those receiving routine hospital care [5]. Malawi has shown similar success in decentralizing care and allowing non-physician clinicians to initiate ART [6]. In Kenya a clinical trial evaluated whether the ART management could be partially shifted from health care workers to persons living with HIV/AIDS who would monitor patients within the community [7]. This showed similar clinical outcomes but with fewer clinic visits. No studies have estimated the cost differences between the two approaches using primary data.

In 2010, the South African government revised its HIV treatment guidelines for the first time since the program launched in 2004. Among a host of changes to drug regimens, eligibility criteria, and monitoring protocols, the revised guidelines included the following two objectives: to “enable nurses to initiate ARVs for treatment and prevention” and to “enable primary health care facilities to initiate, manage, monitor, and refer patients.” Justification for this change included a recently published randomized trial set in South Africa demonstrating that, under the carefully regulated conditions of a clinical trial, patients initiated on ART by doctors, but monitored by nurses, at primary health care clinics achieved similar treatment outcomes as those managed solely by doctors [8].

Prior to the 2010 guideline revision, a number of interventions to shift treatment delivery to lower level facilities and from doctors to nurses had already been piloted in routine care settings. Among these was a strategy of “down referring” stable, adult ART patients from a central hospital to nearby primary clinics—the same strategy tested by the randomized clinical trial in South Africa, but in a routine care setting—pioneered by the Gauteng Province Department of Health and Right to Care, a South African nongovernmental organization supported primarily by the United States President's Emergency Plan for AIDS Relief. To evaluate the implications of this down-referral strategy for treatment outcomes and costs, we conducted a cost-effectiveness analysis of the treatment program at the Themba Lethu Clinic in Johannesburg and a nearby primary health clinic serving as a down-referral site.

## Methods

### Ethics Statement

The University of the Witwatersrand and Boston University provided ethical approval of the study. In order to protect the anonymity of the patients, the study was conducted as an unlinked, retrospective analysis of a patient data set that did not contain any individual identifiers.

### Down-Referral Procedures

Under the down-referral strategy evaluated in this study, all patients receive pre-ART care and initiate ART at an accredited, hospital-based HIV/AIDS clinic, which we will refer to as the treatment-initiation site. For patients who are eligible for and accept down-referral, management of their care is moved to a down-referral site, which is a primary health clinic (PHC). PHCs typically provide reproductive, maternal, and child health care, TB treatment, HIV testing, and other basic services but are not typically accredited to provide ART.

To be eligible for down-referral, patients must have been on ART for at least 11 mo; they must have no opportunistic infections, a CD4 cell count >200 cells/mm^3^, and a stable weight as reflected by <5% weight loss between the last three visits; and their latest HIV viral load within the last 10 mo must be undetectable (<400 copies/ml). The treatment-initiation site involved in this study performs the first routine viral load test after 4 mo on treatment, and the second at 10 mo, establishing the time intervals used in the down-referral criteria. Treatment-initiation site patients who meet these criteria are invited (but not required) to transfer to a down-referral site. For those who accept down-referral, the treatment-initiation site doctor then writes a prescription for 6 mo of ARVs. Down-referred patients are dispensed a 2-mo supply of ARVs at the treatment-initiation site and make an appointment at the down-referral site for 2 mo later. All subsequent ARV pickups occur at the down-referral site. At both the treatment-initiation and down-referral sites, stable patients are scheduled for medication pickups every 2 mo. Treatment follows South African national guidelines, which during the study period called for a first-line regimen of stavudine, lamivudine, and nevirapine or efavirenz and routine CD4 counts and viral load tests every 6 mo.

The main difference between the routine monitoring conducted at the down-referral site and the treatment-initiation site is the cadre of clinical staff involved. While the treatment-initiation site relies on medical consultations with doctors, patients at the down-referral site see only PHCNs, professional nurses with a qualification in primary health care. Down-referred patients have a nurse consultation at every routine visit (every 2 mo), while patients at the treatment-initiation site have a doctor consultation only every 6 mo. At each visit, the PHCN asks whether the patient has had any unexplained weight loss, experienced symptoms related to hyperlactatemia, and/or visited any other medical facility since the last appointment. If none of these events has occurred, the patient collects a 2-mo supply of medications and is scheduled for an appointment 2 mo later. Otherwise, if any of these events has occurred, the PHCN conducts a full consultation. The PHCN may prescribe or change non-ARV medications and renew existing ARV prescriptions but cannot alter the patient's original ARV prescription. After a full consultation, the PHCN can continue the patient on the standard drug collection and visit schedule (i.e., every 2 mo), treat the patient and request a follow-up visit before the next scheduled visit, or up-refer (return) the patient to the treatment-initiation site. The PHCN also does a routine blood draw 4 mo into each 6-mo prescription cycle, with samples processed for viral load, CD4 count, full blood count, alanine aminotransferase, aspartate aminotransferase, and, depending on drug regimen, cholesterol, triglycerides, creatinine, and/or lactates. Based on the results of these tests, the PHCN can renew the patient's ARV prescription for another 6 mo, change any concomitant non-ARV medications, draw blood again to confirm the laboratory findings, and/or up-refer the patient immediately. Both sites performed the same routine monitoring tests every 6 mo, but doctors at the treatment-initiation site were allowed to order additional laboratory tests if required, whereas nurses at the down-referral site had a restricted list of laboratory tests available and had to up-refer a patient if non-routine tests were indicated.

Patients who are up-referred to the treatment-initiation site remain under treatment-initiation site care until a treatment-initiation site doctor recommends (re-)down-referral. At both sites, a patient who is more than 3 mo late for the next scheduled visit is classified as lost to follow up.

### Study Sites

The treatment-initiation site in this study is the Themba Lethu Clinic (TLC) of Helen Joseph Hospital in Johannesburg, a large public sector ART clinic at a secondary hospital. The site, which has been described in detail elsewhere [9], started providing ART in April 2004 and has since initiated more than 18,500 patients on treatment. Treatment is initiated and managed by doctors, as required by the treatment guidelines in place in South Africa until 2010. The clinic is sponsored by the Gauteng Province Department of Health, with additional support from the United States President's Emergency Plan for AIDS Relief.

Due to increasing patient numbers and shortages of human, financial, and infrastructural resources, TLC began down-referring to PHCs within its patient catchment area in 2008. Its first down-referral site was Crosby Clinic, a PHC of the City of Johannesburg roughly 3 km from TLC. Crosby Clinic established a down-referral wing that has its own ART waiting area, consultation rooms, and pharmacy and operates independently of other PHC departments. It can therefore be closely integrated with the treatment-initiation site and avoid the long patient waiting times that are characteristic of many PHC facilities. Because it is located near the treatment-initiation site, patient transport costs are similar between the two facilities.

The treatment-initiation and down-referral sites make use of the same electronic patient management and data system, Therapy Edge-HIV. The system transfers patient records between the treatment-initiation and down-referral sites when a patient is down- or up-referred, alerts the provider if up-referral is required, and automatically transfers files and schedules appointments as needed.

### Sample Selection and Matching

We conducted a secondary analysis of routinely collected data from a cohort of patients followed in a routine care setting. As the intervention had already been implemented, we were unable to randomize to limit confounding. Instead, we used a quasi-experimental design in which we matched down-referred patients (down-referral group) to non-down-referred patients (treatment-initiation group) to attempt to create similar populations at the time of eligibility for down-referral. We included all ART patients down-referred from TLC to Crosby Clinic between 1 February 2008 and 1 January 2009. Down-referred patients were matched to not down-referred patients 1∶3 based on gender, age (18–24.99, 25–29.99, 30–39.99, 40–49.99, and ≥50 y), time on ART (in 6-mo intervals), ARV regimen at study eligibility, and CD4 count at study eligibility (0–99 100–199, 200–349, and ≥350 cells/mm^3^). Matching was accomplished though propensity scores [10],[11]. We allowed treatment-initiation site patients to be used as a match more than once, but not within the same 6-mo time interval. Patients were excluded from both groups if they were (1) down-referred to a site other than Crosby Clinic, (2) had <12 mo of potential follow up after down-referral or down-referral eligibility, (3) initiated treatment outside TLC, (4) had a non-standard treatment regimen; or (5) had missing or erroneous medical record data. In the remainder of this paper, “study eligibility” refers to eligibility for either group.

### Data Collection

Methods for data collection and costing have been described previously [12]. Taking the provider's perspective, each study participant's electronic medical record was reviewed, and resource usage data for the 12-mo period following down-referral eligibility were collected. For down-referred patients who returned to the treatment-initiation site during the study period (i.e., were up-referred), resource utilization included resources provided by the treatment-initiation site. Resources captured included ARVs, non-ARV medications, laboratory tests, outpatient visits, infrastructure, equipment and furnishings, data capture, and program management. An outpatient visit at the treatment-initiation site included consultations with both a nurse and a doctor; if a patient also collected medications, an additional pharmacy cost was added to the total visit cost. An outpatient visit at the down-referral site was limited to a consultation with the PHCN, who also dispensed medications. Utilization of fixed resources (e.g., infrastructure and administrative staff) for participants who died or were lost to follow up was prorated based on the number of months the patient remained in care. Unit costs were estimated in 2009 South African rand from service provider price lists and financial information provided by the sites. Costs were converted to US dollars at a rate of R8.40 to US$1.00.

### Data Analysis

Each study participant at both sites was assigned to a single outcome category on the basis of patient status 12 mo after down-referral eligibility. Criteria for assigning outcomes were adapted from previous work in South Africa assessing patient outcomes 12 mo after treatment initiation [12]. To cope with inconsistent timing of clinic visits and laboratory tests, medical record information within a 3-mo window on either side of 12 mo (i.e., 9–15 mo after down-referral eligibility) was used in determining outcomes, as shown in Table 1.

**Table 1:** Criteria for assigning HIV treatment outcomes.

Outcome	Criteria for Assigning Outcome	Comments
Excluded from study	Started treatment prior to public rollout	Patient initiated ART prior to the South African national treatment plan launched in April 2004
	Outside of study period	Down-referred/eligible for down-referral before 1 February 2008 or after 1 January 2009
	Less than 12 mo of potential follow up	Started ART <12 mo prior to data collection or transferred formally to a different site; informal transfers without medical record notation are recognized as loss to follow up
	Non-standard ARV regimen	Patient on a regimen that was not part of the national treatment guidelines during the study
	Missing matching data/erroneous data	Includes patients who do not have a viral load, CD4 count, regimen, weight, age, or gender
No longer in care	Died	Only reported deaths included; deaths never reported to the site are recognized as loss to follow up
	Lost to follow up	≥3 mo late for last scheduled consultation or medication pickup
In care but not responding	Detectable viral load (virologic failure)	Viral load >400 copies/ml in month 9–15
	If no viral load available, insufficient CD4 change (immunologic failure)	CD4 decrease of ≥30% from peak or ≤baseline in month 9–15
In care and responding	Undetectable viral load	Viral load ≤400 copies/ml in month 9–15
	If no viral load available, sufficient CD4 change	CD4 within 30% of peak and >baseline in month 9–15
	If no viral load or CD4 count available, still in care	Default outcome for patients remaining alive and in care but without laboratory results

**Figure 1:**
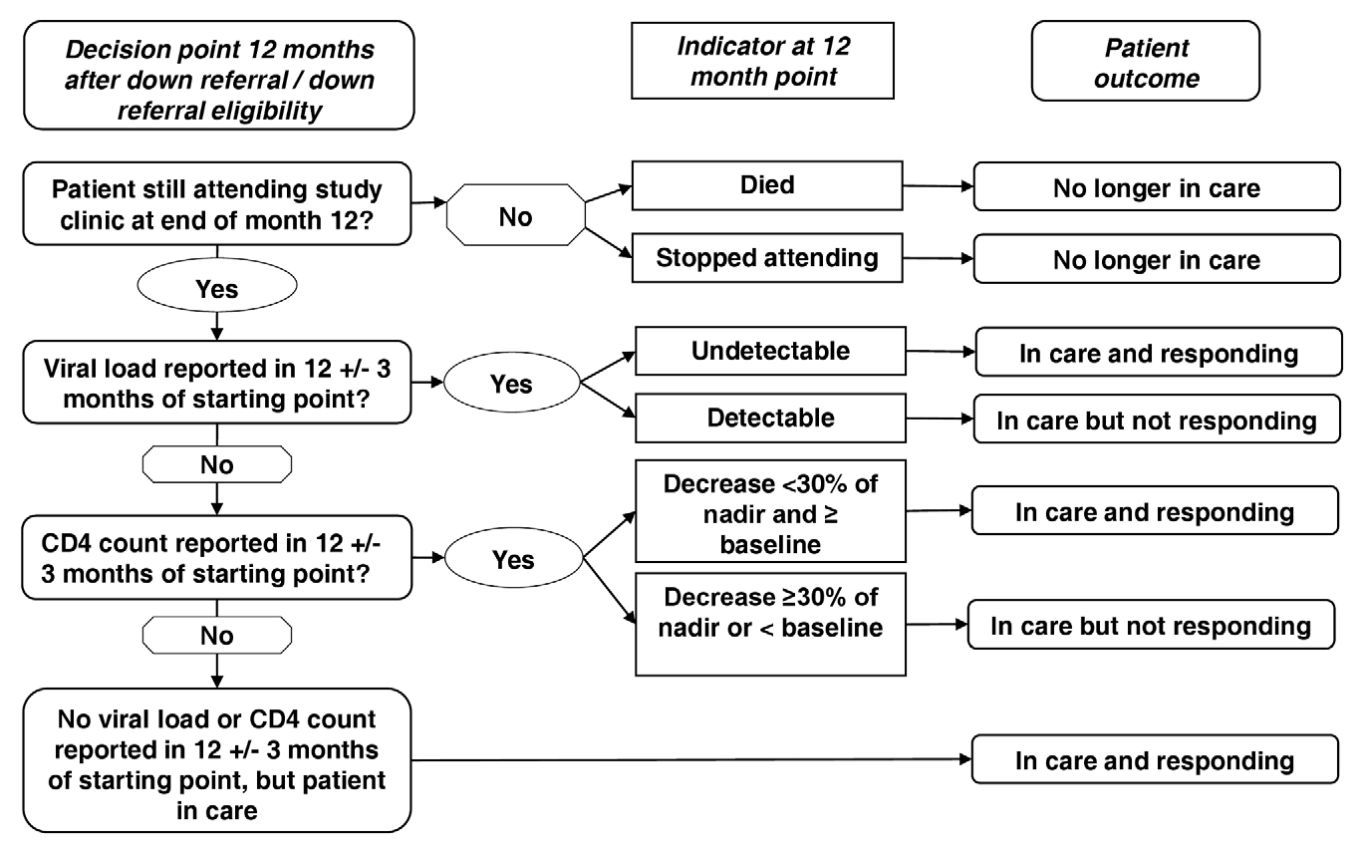
Decision process for assigning HIV treatment outcomes. Patients were placed in a mutually exclusive patient outcome category 12 mo after study enrolment – no longer in care, in care and responding or in care and not responding. Patient outcomes were defined based on the patient's vital status, presence in the clinic, viral load or CD4 count at 12 mo after study enrolment. For those patients alive and in treatment, viral load was the preferred outcome indicator, but in the absence of viral load CD4 count was used and if neither were available then it was assumed the patient was in care and responding based on their presence in the clinic. The diagnostic result closest to 12 mo, but within 3 mo (9–15 mo) was used.

Using these criteria, outcome assignments were made hierarchically, as illustrated in Figure 1. Up-referral—return of a down-referred patient to the treatment-initiation site for monitoring and care—was not considered a discrete outcome. Outcomes for up-referred patients were assigned using the same criteria as for all other patients, as defined in Table 1, and costs incurred at the treatment-initiation site following up-referral are included in the cost per down-referred patient.

Once an outcome was assigned, we estimated a total cost for each patient based on all resources utilized during the 12-mo study period. We estimated an average cost per patient-year in care for each outcome category by site. The cost-effectiveness of the two sites was compared on the basis of average cost per patient remaining in care and responding at the 12-mo point. We created 95% confidence intervals (CIs) around these estimates using bootstrapping with 10,000 replicates [13].

## Results

### Sample Selection and Cohort Characteristics

**Figure 2:**
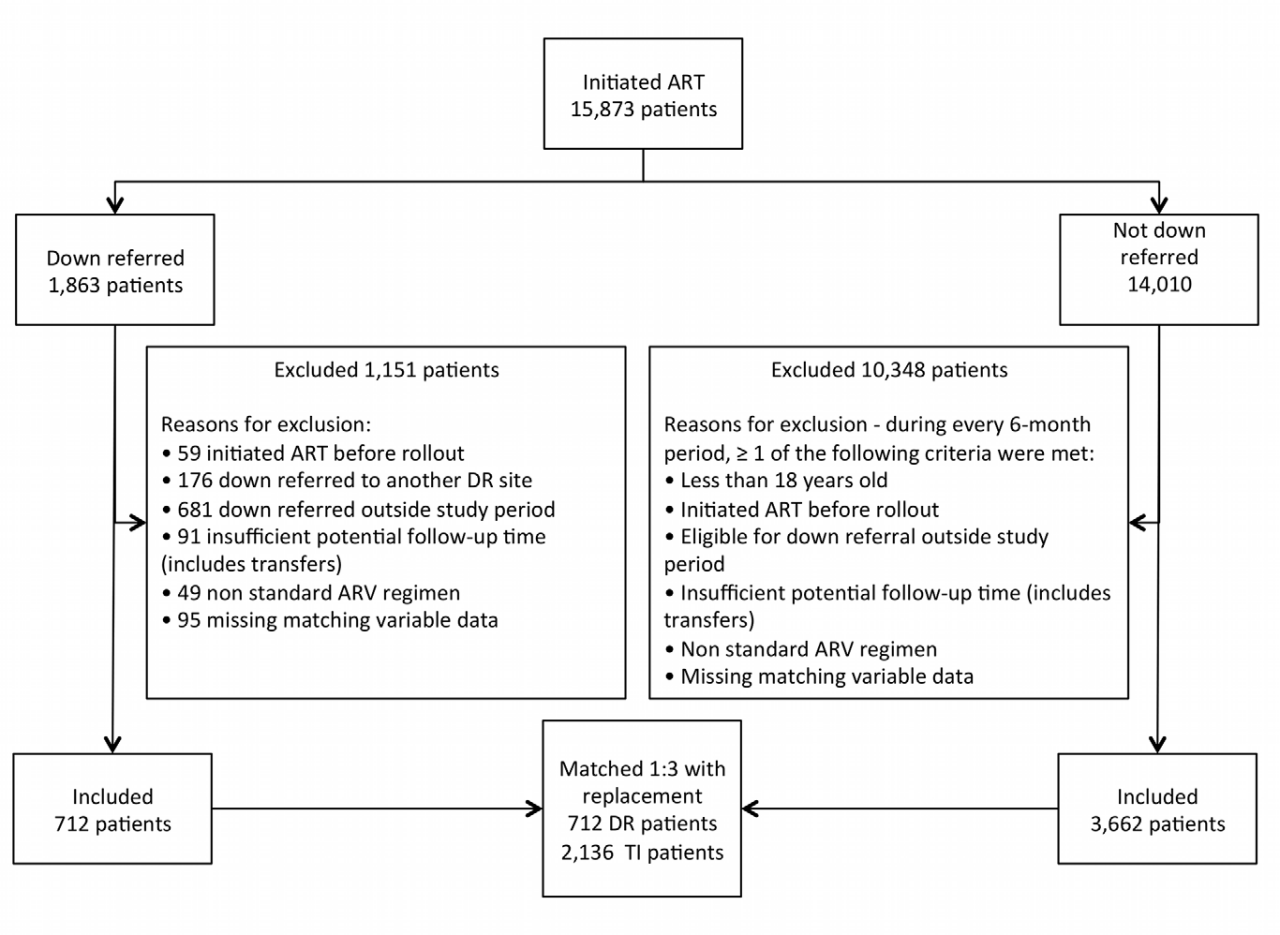
Selection of study participants. All patients initiated on ART at the treatment-initiation site were considered for this study. The patient population was divided into those down-referred and those not down-referred (maintained at the treatment-initiation site). Patients were excluded if they (1) had missing matching variables, (2) were on non-standard ARV regimens, (3) had insufficient potential follow-up time, (4) were eligible/down-referred outside the study period, (5) initiated ART prior to the national rollout, (6) were down-referred to another site (i.e., not the study site), or (7) were <18 y old. Those down-referred patients included in the study were matched 1∶3 with patients maintained at the treatment-initiation site. DR, down-referral; TI, treatment-initiation site.

Selection of patients from each site is illustrated in Figure 2, and study participants are described in Table 2. We enrolled 712 study participants from the down-referral site and 2,136 patients from the treatment-initiation site. There was little difference between the groups in the characteristics on which they were matched. Down-referred patients excluded because of missing data were similar to patients in the down-referral group in terms of age, time on ART, CD4 count, and treatment regimen.

**Table 2:** Characteristics of ART patients at the time of eligibility for down-referral to nurse-managed care.

Variable	Treatment-Initiation Group	Down-Referral Group
**Number**	2,136	712
**Mean age at study eligibility, years (IQR)** a	38.5 (32.7–43.68)	38.5 (33.06–42.03)
**Sex, percent female** a	65.5	65.3
**Median CD4 count at ART initiation, cells/mm^3^ (IQR)** b	94 (36–163)	103 (41–168)
**Median CD4 count at study eligibility, cells/mm^3^ (IQR)** a	397 (309–521)	404 (318–526)
**Mean duration on ART at study eligibility, years (IQR)** a	2.3 (1.8–3.3)	2.3 (1.8–3.3)
**ARV regimen at study eligibility (percent)** a		
Stavudine–lamivudine–efavirenz	64.8	63.8
Zidovudine–lamivudine–efavirenz	27.1	27.1
Other	8.1	9.1

a These characteristics were matched between the two samples.

b Note that 15.4% of participants in the treatment-initiation sample and 11.5% in the down-referral sample did not have baseline CD4 counts reported.

IQR, inter-quartile range.

### Treatment Outcomes

Treatment outcomes are reported in Table 3. Both groups showed very good outcomes 12 mo after down-referral eligibility, with well over 90% of patients remaining in care. The down-referral site experienced fewer deaths and losses to follow up, losing just 2% of its patients during the observation period. As a result, the relative risk of no longer being in care in the down-referral group was markedly lower than in the treatment-initiation group (relative risk = 0.27, 95% CI 0.15–0.49). The adjusted hazard ratio was estimated, but it showed no difference from the relative risk estimate. In total, 122 patients in the down-referral group (17.1%) were up-referred within the 12-mo period, after an average of 6.1 mo at the down-referral site. These participants remained in the down-referral group for this analysis. Most of them met the criteria for “in care and responding” after up-referral and are categorized as such in Table 3.

**Table 3:** HIV treatment outcomes 12 mo after down-referral eligibility.

Outcome	Treatment-Initiation Group (*n* = 1,623)	Down-Referral Group (*n* = 540)	Relative Risk (95% CI)^a^
Total	2,136 (100%)	712 (100%)	—
In care and responding	1,912 (89.5%)	680 (95.5%)	1.07 (1.04–1.09)
In care but not responding	91 (4.3%)	20 (2.8%)	0.66 (0.39–1.06)
No longer in care	133 (6.2%)	12 (1.7%)	0.27 (0.15–0.49)
Died	25 (1.2%)	0 (0.0%)	
Lost to follow up	108 (5.1%)	12 (1.7%)	

a Relative risk of outcome at down-referral site, with treatment-initiation site as reference.

### Resource Utilization

Average resource utilization by patients who remained in care at 12 mo is shown in Table 4, which also provides unit cost estimates for the resources for which unit cost varied by site. Usage of viral loads and CD4 counts varied little between the samples. Down-referred patients made nearly twice as many clinic visits as patients remaining at the treatment-initiation site, but at a cost per visit of less than half. Fixed costs were also much lower at the down-referral site than at the treatment-initiation site.

**Table 4:** Average resource utilization and selected unit costs for the study sample (*n* = 2,160) over 12 mo from the date of down-referral eligibility.

Resource	Treatment-Initiation Site	Down-Referral Site
**Average utilization per patient-year in care**		
Treatment-initiation site visit	4.4	1.5
Down-referral site visit	0.0	5.6
CD4 count	1.6	1.5
Viral load test	1.6	1.4
**Unit costs** a		
Average cost per outpatient visit, US$	14	7
Average fixed cost per patient per month, US$	98	59

a Costs were converted to US dollars at a rate of R8.40 to US$1.00.

### Costs and Cost-Effectiveness

The average cost per patient by outcome, and the breakdown of total cost by major cost components for patients who remained in care and responding at 12 mo, are reported in Table 5. For a patient who remained in care and responding for the 12 mo following down-referral eligibility, treatment at the down-referral site cost an average of US$59 (95% CI US$49–US$70, *p*<0.001) per year less than at the treatment-initiation site, a savings of 11%. The down-referral site spent less on every component of cost except ARVs. Differences in non-ARV drug and lab test costs can be attributed to the greater latitude doctors have in prescribing drugs and diagnostics beyond what is mandated by guidelines. Despite the larger number of clinic visits per patient-year by down-referral patients, the down-referral site still saved money on outpatient visits because of its much lower unit cost per visit.

**Table 5:** Average cost per patient, by HIV treatment outcome and cost component.

Outcome or Cost Component	Cost, in US Dollars	
	Treatment-Initiation Patients	Down-Referral Patients
**Outcome, mean (standard deviation)**		
All patients in group	539 (141)	486 (98)^a^
In care and responding	551 (128)	492 (88)^a^
In care but not responding	589 (163)	481 (97)^a^
No longer in care	330 (147)	175 (103)^a^
**Cost component, value (proportion of total)** b		
Drugs—ARV	237 (43%)	262 (53%)
Drugs—non-ARV	16 (3%)	7 (1%)
Lab tests	120 (22%)	101 (21%)
Outpatient visits	80 (15%)	60 (12%)
Fixed costs	98 (18%)	62 (13%)
Total	551 (100%)	492 (100%)

Costs are given in 2009 US dollars, converted at a rate of R8.40 to US$1.00.

a *p*<0.001 for difference between treatment-initiation and down-referral patients.

b Only patients who were still in care and responding at 12 mo were included.

Using the data in Tables 3 and 5, we estimate that the down-referral site spends an average of US$509 (95% CI US$500–US$519) to produce a patient who is in care and responding 1 y after down-referral, taking into account the resources invested in those who do not respond, die, or are lost to follow up, and including the costs of treatment-initiation site treatment for those up-referred. The treatment-initiation site spends an average of US$602 (95% CI US$592–US$612). For this pair of sites in South Africa, down-referral to a PHC is the cost-effective strategy for eligible patients.

## Discussion

There is general agreement in the literature and in reports from international agencies that some combination of task-shifting from higher to lower level care providers and decentralization from higher to lower level facilities is essential if targets for HIV/AIDS treatment access in resource-constrained countries are to be met in the face of still-rising patient numbers and declining donor budgets [4]. In this study comparing the outcomes and costs of treatment provided by PHCNs at a PHC with those of treatment provided by doctors at a hospital-based clinic, we found that, for those patients eligible for down-referral, the down-referral site achieved better outcomes at consistently lower costs than the treatment-initiation site.

Both sites produced very good outcomes for the patients sampled. To some extent this is not surprising, as only stable patients who had been on ART for at least 11 mo were eligible for the study. The relatively large number of patients who die, are lost to follow up, or fail treatment in the first year on ART were thus deliberately excluded. Even considering the strict inclusion criteria, however, outcomes at the down-referral site, where almost 95% of patients remained in care, were exceptional.

A major impetus for task-shifting and decentralization, in South Africa and elsewhere, is cost. There is a clear expectation that moving treatment to lower level clinical staff and facilities will cost less than the status quo. In this study, we observed a cost reduction of roughly 11% per patient-year in care. Although this percentage may be smaller than hoped for, the scale of the treatment program in South Africa makes the potential for absolute savings large. South Africa currently has nearly one million patients on ART [14]. If even a quarter of these could be down-referred, and if the cost difference we found is applicable to sites across the country, the estimated difference in cost of US$59 per patient per year would result in program-wide savings of more than US$14 million per year, enough to support an additional 25,000–30,000 patients on ART per year. Using electronic data from the treatment-initiation site where we conducted the study, we estimated that roughly 43% of all patients who initiated ART in the first half of 2008 met eligibility criteria for down-referral by the end of 2009, suggesting that savings could exceed US$25 million per year. These savings would offset roughly 10%–15% of the cost of adding the 400,000 new patients per year called for by the country's national plan for achieving universal treatment access [15].

### Limitations of the Study

Our study had a number of limitations. It looked at only one pair of sites, which may or may not be representative of other facilities in South Africa. Both were high volume, well-resourced, urban sites in one of South Africa's wealthier provinces. Because the treatment-initiation and down-referral sites in the study were located close together, most patients did not face different obstacles in accessing the sites, such as higher transport costs. This is unlikely to be the case in many settings. Both sites were government health facilities and followed the prescribed treatment regimens and monitoring algorithms as outlined in the national treatment guidelines. In these respects, the study sites were comparable to the many public sector treatment sites within South Africa. Both sites did receive some external donor support, however, and our findings might not pertain to under-resourced sites. The generalizability of our findings to other African countries may also be limited because of the diverse conditions under which care and treatment are offered across sub-Saharan Africa. For example, South Africa relies on doctors, rather than clinical officers, to provide routine care and has access to a wider range of diagnostics (e.g., viral load tests) than do many other countries.

A well-designed randomized clinical trial usually produces the most unbiased outcome estimates (internal validity), but may have a low degree of external validity, or relevance to the real world [16]. While using observational data generated through routine practice may have allowed some bias in our study, it also strengthened the relevance of our findings. In addition, by matching participants in our study, we reduced the potential for any strong bias. Still, while we matched down-referred and non-down-referred patients on known and measured factors affecting treatment outcomes, there is the potential that our populations differed with respect to some unmeasured factor. In order to determine the sensitivity of our results to changes in patient outcomes as a result of such a factor we varied (decreased) the proportion of patients in care and responding in the down-referred arm. The proportion of patients in care and responding would have to drop from 96% to 77% in this arm, with no changes to the initiation-site arm, for the costs per patient in care and responding to be equal. If we take the more likely scenario of equivalent outcomes as seen in the CIPRA clinical trial [9], the cost per nurse-managed patient in care and responding would still be lower than the cost of those managed by doctors ($509 versus $602), and nurse-managed care would therefore still be the preferred choice.

The study reflects treatment guidelines that were in effect until 2010, including the use of stavudine in first-line treatment. In the revised guidelines, new ART patients and those experiencing stavudine toxicities will be prescribed tenofovir. The much greater tolerability of tenofovir may alter the relative effectiveness of doctors and nurses in managing ART patients. While we would expect this to make it even easier for nurses to manage treatment, we cannot be certain. We also cannot evaluate the extent to which the relatively low patient volumes seen at the down-referral site affected the quality of care delivered. Very large down-referral sites, and those that have exceeded their service delivery capacity, may achieve poorer patient outcomes than those we observed. Finally, as has been noted in recent publications, the rate of reported “loss to follow up” from any one treatment facility may overstate actual patient attrition from treatment, as many patients who are reported as lost have actually re-started treatment somewhere else [17]. If this phenomenon was more common at the treatment-initiation site in our study than at the down-referral site, the actual difference in outcomes between our two sites may be less than we observed.

### Conclusions

In addition to the financial cost savings estimated in this study, transferring patients to nurse-managed, primary-level clinics has the additional advantage of freeing up the time and resources of more highly trained doctors and well-equipped facilities to focus on patients who are not responding to treatment or have other complications. Task-shifting allows more health care workers to provide ART care, and this in turn increases the treatment coverage available to meet the large unmet need. Although South Africa faces a shortage of both doctors and nurses, the scarcity of doctors is greater [18], and this scarcity may not be fully reflected in the salary differential between the two job grades. The financial cost-effectiveness analysis presented here thus does not capture the full opportunity costs of the intervention evaluated.

The national treatment guidelines adopted in 2010 allow nurses to initiate, as well as manage, ART, under a model known as NIMART (Nurse-Initiated and Managed ART). This policy change was based on modeled estimates of the cost savings from task-shifting but with little empirical evidence confirming that these savings will indeed be realized. This study provides strong evidence that at least part of the NIMART model—the management of stable patients by PHCNs—will reduce overall treatment program costs in South Africa, without compromising patient outcomes.

## Abbreviations

ART: antiretroviral therapy
ARV: antiretroviral drug
CI: confidence interval
PHC: primary health clinic
PHCN: primary health care nurses
TLC: Themba Lethu Clinic
